# Data confirming murine erythrocyte opsonization and oxidative damage and live microscopic analysis of oxidatively damaged erythrocyte uptake by mast cells

**DOI:** 10.1016/j.dib.2018.08.192

**Published:** 2018-09-13

**Authors:** Priyanka Sharma, Niti Puri

**Affiliations:** School of Life Sciences, Jawaharlal Nehru University, New Delhi, India

## Abstract

The data in the present article are related to research article (doi: https://doi.org/10.1016/j.imlet.2018.04.002) [1]. The data describes the detailed immunization protocol for generating polyclonal antisera to murine erythrocytes in rat. The rat anti-mouse erythrocyte serum is then tested for its ability to bind and opsonize murine erythrocytes. Second set of data confirms the oxidative damage to murine erythrocytes by treatment with different dose of the *tert*-butyl hydroperoxide (*t*-BHP) on the basis of phosphotidylserine externalization by murine erythrocytes as well as measurement of reactive oxygen species (ROS) formation in *t*-BHP treated erythrocytes. Third set of data depicts lack of mast cell degranulation in the form of β- hexosaminidase release in response to co-incubation of mast cell with normal and oxidatively damaged erythrocytes. Lastly, the uptake of oxidatively damaged erythrocytes by resting and activated RBL-2H3 mast cells is shown by live cell imaging using confocal microscope.

**Specifications table**

**Table:** 

Subject area	*Immunology*
More specific subject area	*Cellular immunology, Phagocytosis by mast cells*
Type of data	*FACS histograms, graphs, images, video*
How data was acquired	*Flow cytometer (BD FACS calibur was used to acquire the data and analyzed on CellQuest Pro software), microplate reader (spectramax M2e by Molecular Devices), fluorescence images (Nikon Eclipse-Ti flourescence microscope), live cell imager Andor Spinning Disk Confocal microscope (Nikon Eclipse TiE, Software-Andor iQ 2.7)*
Data format	*Analyzed*
Experimental factors	*Pre-treatment of erythrocytes with t-BHP to induce oxidative damage*
Experimental features	*Generation of anti-mouse RBC sera and its confirmation, induction of oxidative damage, its confirmation and co-incubation of mast cells and erythrocytes for exocytic response and live cell confocal microscopy*
Data source location	*not applicable*
Data accessibility	*Data is within present article*
Related research article	*P. Sharma, N. Puri, A new role for mast cells as scavengers for clearance of erythrocytes damaged due to oxidative stress, Immunology Letters. 2018 Jul;199:23-35.*
	doi: 10.1016/j.imlet.2018.04.002.

**Value of the data**

• This data describes the detailed immunization protocol and confirms generation of rat anti-mouse RBC serum, which can be a valuable tool for immunological research involving opsonized RBCs.
• The present data could be helpful for further studies with *t-*BHP induced oxidatively damaged erythrocytes.
• This data explains cellular and morphological changes in mast cells with time in resting and activated states and their interaction with normal or oxidatively damaged erythrocytes.
• The present data provides a platform to further explore the detailed underlying mechanism of phagocytosis of erythrocytes by mast cells.

## 1 Data

**Fig. 1:**
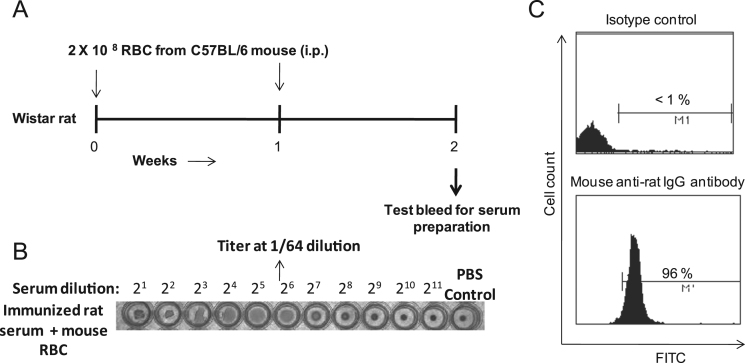
**Generation of rat anti-mouse RBC serum and opsonization of mouse erythrocytes.** (A) Wistar rat was immunized intraperitoneally for 2 weeks with 2 × 10^8^ mouse erythrocytes (per dose once a week) and test bleed was taken 1 week after last immunization for serum isolation. (B) hemagglutination titer was checked from the rat sera at various dilutions. (C) Opsonization of murine erythrocytes with rat anti-mouse RBC sera was confirmed with FITC labelled mouse anti-rat IgG antibody.

**Fig. 2:**
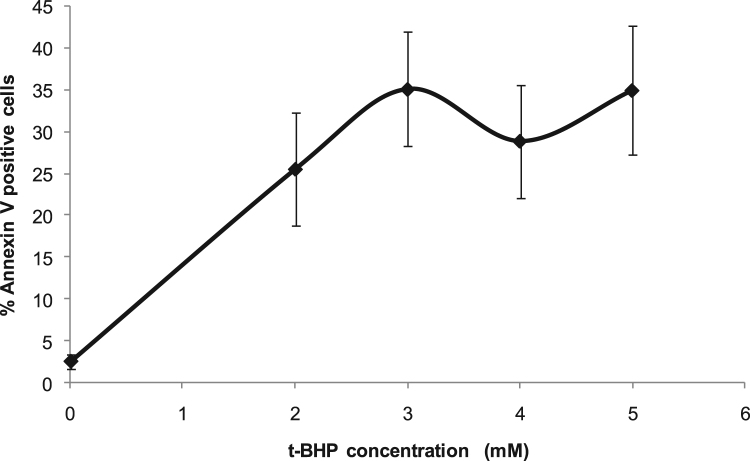
**Phosphatidyl serine externalization in erythrocytes treated with different doses of*****t*****-BHP.** Mouse erythrocytes were treated or not with different doses of tert-butyl hydroperoxide (*t*-BHP) as described in material and methods. Treated and control erythrocytes were stained with APC conjugated annexin V and were analyzed for PS externalization on BD FACSCalibur (*n* = 3).

**Fig. 3:**
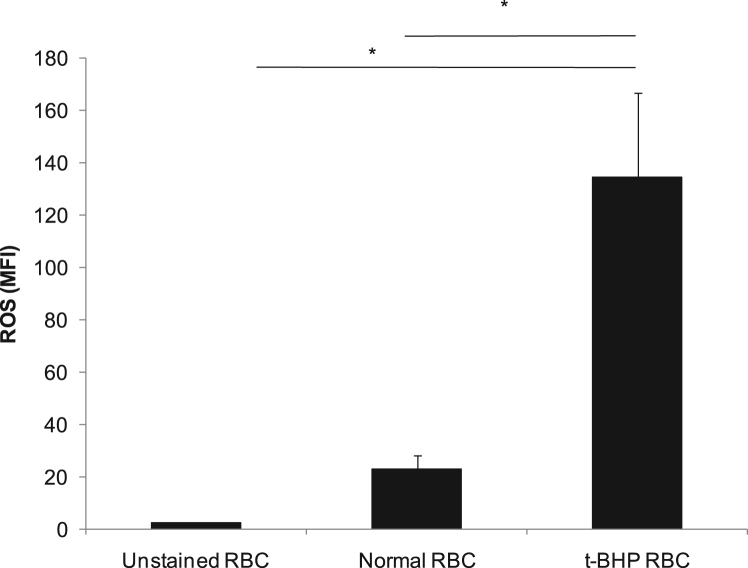
**Generation of Reactive Oxygen Species (ROS) in normal and*****t*****-BHP treated erythrocytes.** Reactive Oxygen Species (ROS) was measured in normal or *t*-BHP treated oxidatively damaged erythrocytes by staining with CMH_2_DCFDA. The mean fluorescence intensities (MFI) were analyzed from the erythrocytes using flow cytometry on the basis of resultant fluorescent products generated due to ROS in the erythrocytes. MFI due to ROS generation was compared in unstained, normal and *t*-BHP treated erythrocytes and plotted as bar graph (* *p* ≤ 0.05, *n* = 3).

The first data set shows detailed protocol to generate anti-mouse RBC serum in Wistar rat (Fig. 1(A)). The antibody titer in rat serum was checked by hemagglutination test and found to be 1:64 (Fig. 1(B)). The opsonization of murine erythrocytes with rat anti-mouse RBC serum (at 1:64 dilution) was confirmed using anti-rat IgG antibody by flow cytometry (Fig. 1(C)). Data showing the optimized dose of *t-*BHP, an oxidative stress inducing agent [2], [3], based on PS externalization (Fig. 2), and the level of ROS formation by normal and *t-*BHP induced oxidatively damaged erythrocytes (Fig. 3) are shown.

**Fig. 4:**
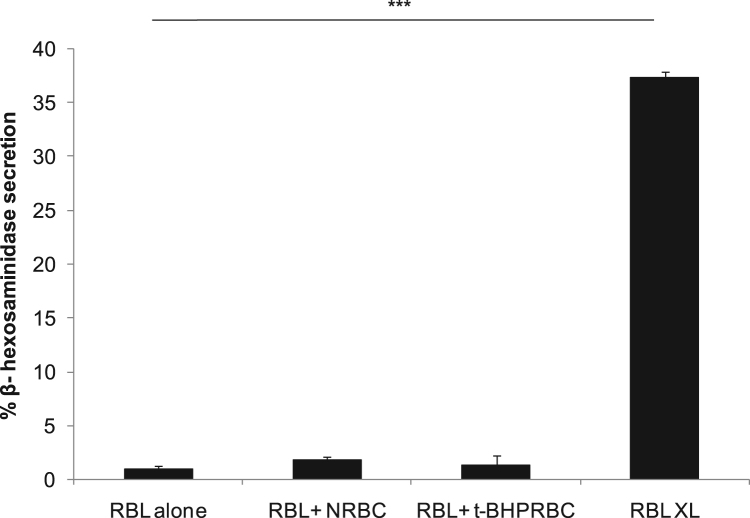
**Mast cell secretion with normal,*****t*****-BHP treated erythrocytes and FcεRI receptor crosslinking.** Percent secretion of β- hexosaminidase by mast cell alone, co-incubated with normal, *t*-BHP treated oxidatively damaged erythrocytes and IgE crosslinked mast cells was analyzed as described in material and methods. Bar graph was plotted on the basis of β- hexosaminidase secretion by mast cell in the presence or absence of normal or *t*-BHP treated erythrocytes and compared with secretion from IgE crosslinked activated mast cells (***P ≤ 0.001, *n* = 3).

**Fig. 5:**
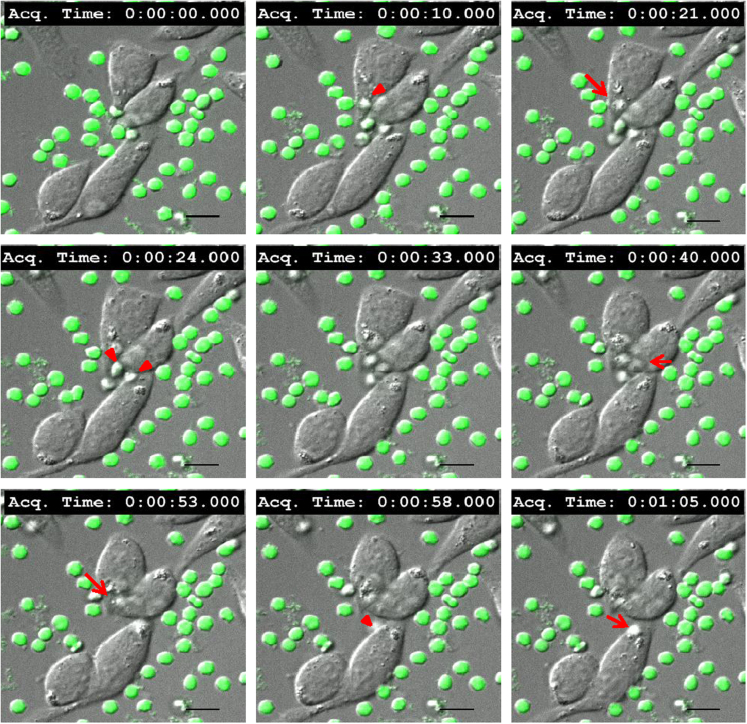
**Live cell imaging of resting mast cells co-incubated with oxidatively damaged eythrocytes.** RBL mast cells were cultured on a glass coverslip overnight and incubated with CFSE-labeled oxidatively damaged erythrocytes (Green) at 37 °C (MC to RBC ratio 1:250). Images were taken every minute upto 90 min continuously under fluorescence microscope and representative images from specific time point were compiled and merged. Arrow heads indicates mast cell-erythrocyte interaction; Arrows indicating uptake of erythrocytes by mast cell (magnification 60×; scale bar, 10 µm).

**Fig. 6:**
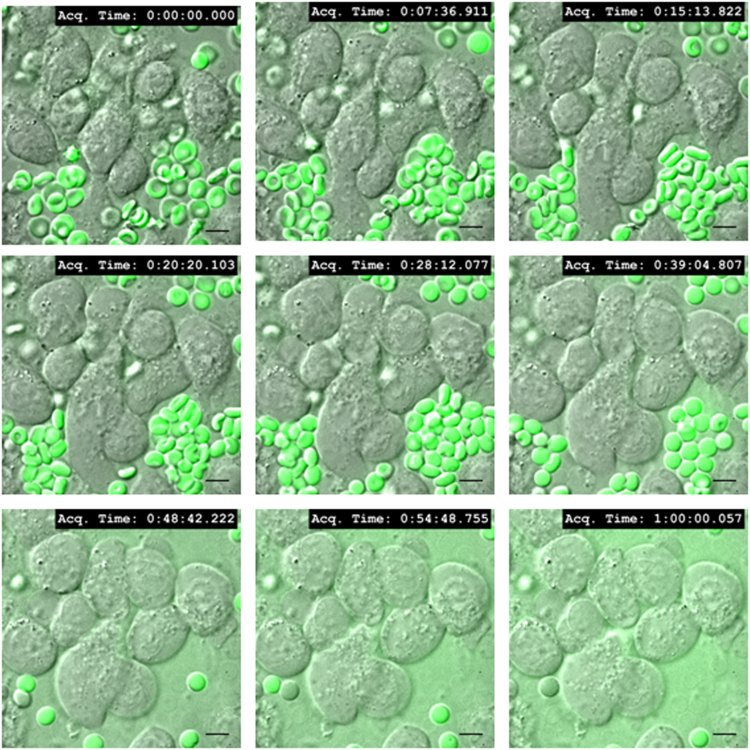
**Live cell imaging of activated mast cells co-incubated with normal eythrocytes.** RBL mast cells were cultured on a glass coverslip and sensitized with IgE after 4 h. After 18 h of seeding CFSE labeled normal erythrocytes (Green) were co-incubated with activated mast cells (simultaneously induced by FcεRI receptor crosslinking) at 37 °C and 5% humified CO_2_ (MC to RBC ratio 1:250). Images were taken every 5 s for upto 60 min continuously under fluorescence microscope and representative images from specific time point were compiled and merged. (magnification 100×; scale bar, 10 µm).

**Fig. 7:**
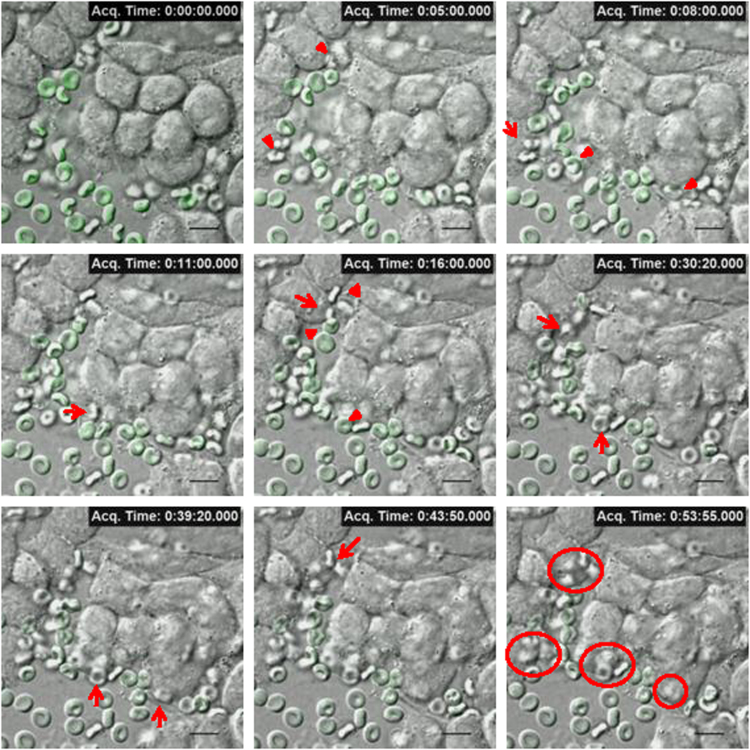
**Live cell imaging of activated mast cells co-incubated with oxidatively damaged eythrocytes.** RBL mast cells were cultured on a glass coverslip and sensitized with IgE after 4 h. After 18 hours of seeding CFSE labeled oxidatively damaged erythrocytes (Green) were co-incubated with activated mast cells (simultaneously induced by FcεRI receptor cross-linking) at 37 °C and 5% humified CO_2_ (MC to RBC ratio 1:250). Images were taken every 5 s for upto 60 min continuously under fluorescence microscope and representative images from specific time point were compiled and merged. Arrow heads indicates mast cell-erythrocyte interaction; Arrows indicating uptake of erythrocytes by mast cell and circle is highlighting/showing engulfed erythrocytes inside mast cells (magnification 100×; scale bar, 10 µm).

Mast cell secretion of β- hexosaminidase due to direct interaction with normal and oxidatively damaged erythrocytes is shown in Fig. 4. RBL mast cells were treated with anti DNP-IgE and allergen (DNP-BSA) for FcεRI receptor crosslinking (XL) [4] as a control. Live cell fluorescence microscopy data was collected by incubating erythrocytes with resting or activated RBL-2H3 mast cells, at 37 °C. Images of resting mast cells co-incubated with oxidatively damaged erythrocytes were captured every minute for 90 min and compiled as video (movie M1). Images of activated mast cells were captured every 5 s for 60 min and compiled as video, when incubated with normal (movie M2) and oxidatively damaged erythrocytes (movie M3). Representative snapshots of uptake of oxidatively damaged erythrocytes by resting mast cells (Fig. 5), and normal (Fig. 6) and oxidatively damaged erythrocytes (Fig. 7) by activated mast cells are shown.

Supplementary material related to this article can be found online at https://doi.org/10.1016/j.dib.2018.08.192.

The following is the Supplementary material related to this article Movie M1, Movie M2, Movie M3.

Movie M1 Live cell imaging of resting RBL mast cells co-incubated with CFSE labeled oxidatively damaged erythrocytes (mast cell to erythrocytes ratio 1:250). Images were acquired every minute upto 90 min at 37 °C, 5% humidified CO_2_ and compiled as visual.

Movie M2 Live cell imaging of activated RBL mast cells co-incubated with CFSE labeled normal erythrocytes (mast cell to erythrocytes ratio 1:250). Images were acquired every 5 s upto 60 min at 37 °C, 5% humidified CO_2_ and compiled as visual.

Movie M3 Live cell imaging of activated RBL mast cells co-incubated with CFSE labeled oxidatively damaged erythrocytes (mast cell to erythrocytes ratio 1:250). Images were acquired every 5 seconds upto 60 minutes at 37^o^C, 5% humidified CO_2_ and compiled as visual..

## 2 Experimental design, materials and methods

### 2.1 Opsonization of murine erythrocytes

Wistar rats were maintained at Central Laboratory Animal Resources-JNU, New Delhi as per CPCSEA guidelines [JNU Institutional Animal Ethical Committee clearance (IAEC code 26/2014)]. Rat was immunized as shown in Fig. 1(A). Serum was isolated and titer checked by hemagglutination test by serial dilution using 0.5% of RBC aliquots per well. Dilution of 1:64 was obtained as optimum titer for anti-mouse RBC sera. 100 million mouse erythrocytes were incubated with rat anti-mouse erythrocyte sera at 1:64 dilution, 37 °C for 30 min in continuous rotation of 20 RPM in Hybridization incubator shaker (Amerex instruments, Inc. model HS-111). Opsonization was confirmed using FITC conjugated anti-rat IgG antibody (0.2 µg) and analysis on BD FACSCalibur flow cytometer using CellQuest Pro software (BD Biosciences).

### 2.2 Annexin V staining

Murine (C57BL/6) erythrocytes were pretreated *in vitro* with *tert*-butyl hydroperoxide (*t*-BHP, 3 mM) (Sigma-Aldrich, MO, USA) at 37 °C for 60 min in PBS [2]. Erythrocytes were stained with APC conjugated Annexin V (5 ng) (#640920, Biolegend, San Diego, CA, USA), washed with annexin binding buffer and analyzed on BD FACSCalibur flow cytometer as described earlier [5].

### 2.3 Intracellular ROS measurement in erythrocytes

Normal or *t*-BHP treated erythrocytes (1 million) were incubated with 5 μM CM-H_2_DCFDA (chloromethyl derivative of 2′, 7′-dichlorodihydrofluorescein diacetate) at 37 °C for 30 min as described previously [5]. The samples were analyzed on FL-1 channel with BD FACSCalibur.

### 2.4 Secretion assay of RBL-2H3

RBL-2H3 cells were sensitized with anti DNP IgE (TIB-142 sup), activated with DNP-BSA, and percent secretion was calculated as the percentage of total β-hexosaminidase activity released in the supernatant as described earlier [6].

### 2.5 Live cell imaging

0.2 Million RBL-2H3 cells were cultured overnight in RBL complete medium [1] on 35 mm petridish containing live cell imaging culture chamber (in vitro Scientific, USA). After 18 h, CFSE labeled normal or oxidatively damaged erythrocytes were co-incubated with resting or activated mast cells, in 5% CO_2_ supply for 90 and 60 min respectively. Images were captured continuously at specific time lapse with Andor Spinning Disk Confocal microscope (Nikon Eclipse TiE, Software-Andor iQ 2.7) and analyzed on NIS-Element software.

### 2.6 Statistics

Results from at least three independent experiments are represented as mean ± SEM. Student׳s *t*-test using Microsoft PowerPoint 2007 was performed, and a *p* value of less than 0.05 was considered as statistically significant.
